# Bullying victimization, physical inactivity and sedentary behavior among children and adolescents: a meta-analysis

**DOI:** 10.1186/s12966-020-01016-4

**Published:** 2020-09-11

**Authors:** Antonio García-Hermoso, Ignacio Hormazabal-Aguayo, Xavier Oriol-Granado, Omar Fernández-Vergara, Borja del Pozo Cruz

**Affiliations:** 1 Navarrabiomed, Complejo Hospitalario de Navarra (CHN)-Universidad Pública de Navarra (UPNA), IdiSNA, Calle de Irunlarrea, 3, Postal Code: 31008 Pamplona, Navarra Spain; 2 Laboratorio de Ciencias de la Actividad Física, el Deporte y la Salud, Universidad de Santiago de Chile, USACH, Santiago, Chile; 3 Facultad de Educación, Universidad Andres Bello, Santiago, Chile; 4 Institute for Positive Psychology & Education, Australian Catholic University, Sydney, Australia

**Keywords:** Physical exercise, Sitting time, Screen time, traditional bullying, Cyberbullying

## Abstract

**Background:**

Physical activity and sedentary behavior are related with psycho-social variables among youth, however its relationship with bullying victimization is unclear. The aim of the study was to clarify the associations between physical activity and sedentary behaviors with bullying victimization among children and adolescents.

**Methods:**

Two independent authors searched in four databases. The studies were selected/included only if participants were children and/or adolescents and the relationship between physical activity and/or sedentary behavior with bullying victimization was reported. Random-effects meta-analyses were used.

**Results:**

A total of 18 cross-sectional studies (including 386,740 children and adolescents, 51.8% females) were reviewed. Our study found that not meeting the physical activity guidelines (Odds Ratio [OR] = 1.14, 95% confidence interval [CI], 1.04 to 1.23) and excessive sedentary behavior (i.e., 2 h per day or more of screen time) (OR = 1.21, 95% CI, 1.14 to 1.28) were associated with 14 and 21% higher bullying victimization, respectively. Consistent associations were also found when we analyzed specific forms of bullying for sedentary behavior, including traditional and cyberbullying.

**Conclusions:**

The present study establishes the first quantitative framework for understanding the influence of physical activity and sedentary behavior on bullying victimization, and lays the groundwork for future studies and interventions aimed to its promotion.

**Trial registration:**

CRD42018099388.

## Introduction

Bullying victimization denotes to the process by which an individual is repeatedly and over time exposed to intentional harmful or negative actions by their counterparts [1]. Bullying can occur in different environments, including but not limited to workplace, community settings, schools and home [2]. Bullying victimization is prevalent across countries worldwide, reaching prevalence rates up to 35% for traditional bullying and 15% for cyberbullying [3]. According to the Centers for Disease Control and Prevention (CDC), bullied youths frequently report high levels of poor school performance, sleep difficulties, loneliness, anxiety, depression [4] and are more likely to commit suicide [5].

Physical inactivity among youth predicts a wide range of health problems that are detrimental to well-being [6]. International guidelines from organizations such as the US Department of Health and Human Services [7] and the World Health Organization [8] recommend that children and adolescents should have at least 1 h or more per day of moderate-to-vigorous physical activity. There is evidence that suggests that, when a child or adolescent spends a large amount of time in a sedentary behavior it relates to a poorer health outcome, being somewhat stronger for television (TV) viewing and other screen behaviors than for total sedentary time [9]. In recent years, the rapid adoption of digital media has displaced the consumption of legacy media such as reading a book or a magazine [10]. The American Academy of Pediatrics recommends no more than 2 h per day of any screen-based activity [11]. In this regard, there is evidence that has shown that spending an excessive amount of time in screen-based activities can have a direct relationship to the development of anti-social behavior and aggression [12] and more negative feelings [13]. Therefore, it is important to understand how bullying status relates to whether youth meet or do not meet the physical activity and sedentary behavior recommendations. Therefore, the purpose of this study was to provide a quantitative analysis on the associations of physical activity and sedentary behavior on bullying victimization among children and adolescents.

## Methods

A systematic review and meta-analysis was conducted according to the Preferred Reporting Items for Systematic Reviews and Meta-Analyses (PRISMA) [14]. The review was registered in PROSPERO (registration number: CRD42018099388).

### Data sources and searches

Two independent authors (IH-A & AG-H) searched PubMed, EMBASE, ERIC and PsycARTICLES, from database inception to May 20th, 2020. Studies were identified by using all possible combinations of the following groups of search terms: (a) “bull” OR “victim” OR “peer relation”; (b) “physical activity” OR “exercise”; and (c) “sedentary” OR “screen time” OR “television” OR “video game” OR “computer”. The complete search strategy is shown in Additional file. Only English articles were included. In addition, the reference lists and related links of retrieved articles were examined to detect studies potentially eligible for inclusion.

### Study selection

Studies needed to meet the following criteria: (a) subjects: children and adolescents aged 6 up to 18 years old; (b) type of study: cross-sectional and prospective cohort studies; (c) exposure: objective or subjective measured of physical activity and/or sedentary behavior; and (d) outcomes: bullying victimization as dependent variable. The first and second author (IH-A & AG-H) independently assessed the electronic search results. Reasons for exclusion of identified studies were recorded.

### Data extraction

The following information was extracted from the studies meeting the selection criteria: country of study, participants (e.g., sex, age), type of bullying (i.e., traditional or cyberbullying), physical activity and sedentary behavior assessment of each study and study results.

### Risk of bias in individual studies

The risk of bias was assessed by *The Quality Assessment Tool for Observational Cohort and Cross-sectional Studies* [15]. This methodological tool is composed of fourteen items rated as “yes”, “no” or “not reported”.

### Data synthesis and analysis

Odds Ratio (OR) and 95% confidence interval (CI) were pooled using random effect models (DerSimonian and Laird) [16] to account for anticipated between-study heterogeneity. This heterogeneity (Cochran’s Q-statistic) was estimated using I^2^, considering I^2^ values of < 25%, 25–50, and > 50% as small, medium, and large amounts of heterogeneity respectively [17].

Publication bias was determined by visual examination of funnel plots (only used for overall adiposity). Also, small-study effects bias was assessed using Egger’s test [18].

Finally, subgroup moderator analyses were conducted to determine whether results differed according to sex, specifics form of bullying and type of sedentary behavior.

All analyses were carried out using the STATA 13.1 Software (Stata Corporation LP, College Station, TX, USA).

## Results

### Literature search

**Fig. 1:**
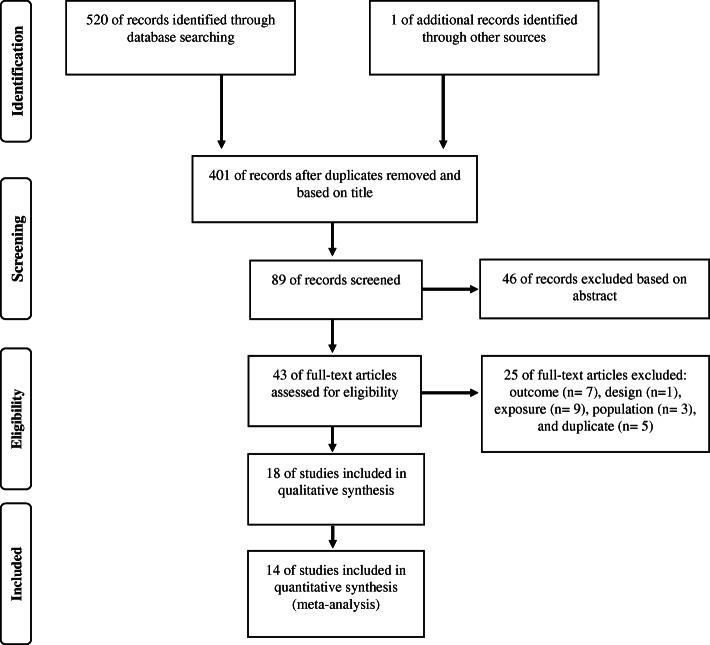
Flow chart for identification of trials for inclusion in the meta-analysis

The electronic search strategy identified 521 studies and, after screening for duplicates, 43 full-text studies were assessed for inclusion after checking titles and abstracts. Finally, 18 studies met the inclusion criteria and were included in the systematic review and 14 in the meta-analysis. The flow diagram showing the number of articles excluded at each stage of the systematic review and meta-analysis is shown in Fig. 1.

### Study characteristics

Table 1 summarizes the characteristics of the 18 included studies. All of them were cross-sectional observational studies, and were published from 2007 to 2020. The studies included 386,740 children and adolescents. Sample sizes ranged from 54 [22] to 237,121 participants [19]. All studies included males and females (51.8% females). Twelve studies included only adolescents (12–18 years old) [19, 20, 24, 27–29, 31, 32, 34–37] and the remaining studies included both children and adolescents.

**Table 1:** Main Characteristics of the Reviewed Studies

Study	Country	Participants/age/female (%)	Types of bullying	PA quality	Sedentary behavior/Screen time assessment	Major findings	Covariates	Methodological appraisal
Alfonso-Rosa 2020 [19]	82 countries	273,121 / 13–15 years / 51.5	T	Self-reported	Self-reported	Excessive sitting time is associated with bullying in the study sample, while physical activity does not.	Age, screen time or physical activity	8
Busch 2013 [20]	The Netherlands	2425 / 11–18 years / 55.0	T	–	TV, internet use and video game playing	Screen time was not associated with bullying	Sex, age, school, year of school, educational level, ethnicity and socioeconomic status	7
Case 2015 [21]	USA	4602 / 13–17 years / 48.3	T	Self-reported	–	Not meeting PA recommendations differs by gender and by the interaction between bullied status and weight status	Weight status, grade, and race/ethnicity	7
Corral-Pernía 2017 [22]	Spain	54 /12–18 years / 55.5	T, CY	Accelerometry	–	Addressing PA practice recommendations not protect against the direct involvement of bullying and cyberbullying	Not mentioned	6
Demissie 2014 [23]	USA	16,410 / 12–18 / 47.8	T	Self-reported	TV and video game playing	Being bullied on school property was associated with lower odds of physical activity among males and higher odds of video game/computer use among females	Race/ethnicity and grade	8
Henriksen 2015 [24]	Denmark	6269 / 11–15 years / 50.8	T	Self-reported	–	The association between exposure to bullying and physical inactivity was significant among students from lower social classes and unclassifiable social class but not among students from higher social classes.	Sex and age	8
Herazo-Berltrán 2019 [25]	Colombia	991 / 7–17 years / 56.7	T	Self-reported	–	The students who did not regularly engage in physical activity had a higher probability of being victims of school harassment	–	7
Hertz 2015 [26]	USA	13,846 / 12–18 years / Not mentioned	T, CY	Self-reported	TV and video game playing	Among male but not female students, having been a victim of both kinds of bullying, as well as having been only in-person bullied, was associated with watching television 3 or more hours per day. While having been a victim of both kinds of bullying was positively associated with using computers 3 or more hours per day among female and male students, having been electronically bullied only also was associated with computer use among male students. Being physically active for at least 60 min/day on 0 of the past 7 days was associated with having been a victim of both kinds of bullying among male students but not female students	Race/ethnicity and grade	8
Katapally 2018 [27]	Canada	44,861 / 13–18 years / 49.4	T, CY	–	Self-reported	Bullying perpetration, victimization, or both are associated with increased multiple screen-time behaviors among youth	Age, ethnicity, weekly disposable income, daylight hours, and weather variables	8
Kelishadi 2015 [28]	Iran	14,880 / 6–18 years/ 49.2	T	–	Self-reported	Prolonged time spent watching TV or using a computer for pleasure may increase the risk of being bullied.	Socioeconomic status, physical activity, sleep hours, family size and body mass index	8
Mendez 2019 [29]	Spain	1248 / 11–18 years / 50.8	T	Self-reported	–	Students who practiced physical activity in the recommended frequency rated as healthy, at least four or more times per week, had higher values in the indicators of aggressiveness than students who practiced with a lower frequency	Not mentioned	7
Merrill and Hanson 2016 [30]	USA	13,583 / 12–18 years / 48.7	T, CY	Self-reported	TV and video game playing	Protective behaviors against bullying victimization and cyberbullying included being physically active. In contrast, students who play video games an average of 3 or more hours per day are at greater risk of being bullied and cyberbullying	Sex, race/ethnicity, and grade	8
Rech 2013 [31]	Brazil	1230 / 11–14 years / 49.3	T	–	Sitting-time	The schoolchildren who reported sedentary habits for more than three hours a day were 55% more likely to be victims	Not mentioned	8
Roman 2013 [32]	USA	7786 / 10–17 years / Not mentioned	T	Self-reported	–	Students who reported being bullied had significantly lower odds of engaging in more than one day of physical activity for 60 min or more	School-level variables	8
Rostad 2018 [33]	USA	15,624 / 12–18 years / 49.6	T, CY	Self-reported	TV and video game playing	Media use was related to experiences of bullying for both male and female students	Race/ethnicity, grade, sexual identity, and substance use—current alcohol use (past 30 days) and current marijuana use (past 30 days)	8
Sampasa-Kanyinga 2020 [34]	Canada	5615 / 14–17 years/ 57.6	T, CY	Self-reported	TV, internet use and video game playing	Meeting the screen time recommendation was associated with lower odds of being a victim	Age, sex, ethnoracial background, subjective socioeconomic status, and body mass index z-score	8
Storch 2007 [35]	USA	100 / 8–18 years / 58.7	T	Self-reported	–	Inverse relation was identified between the reports of peer victimization and the levels of physical activity	Not mentioned	7
Watanabe 2017 [36]	Brazil	95 / 12–14 years/ 49.5	T	Accelerometry	–	Weight-teasing was not related to physical activity	Not mentioned	7

*CY* Cyberbullying; *PA* Physical activity; *T* Traditional; *TV* Television

All included studies used a self-reported questionnaire to assess physical activity and sedentary behavior, except two which assessed physical activity with accelerometers [22, 36]. Most studies defined physical inactivity as less than 60 min of moderate to vigorous-intensity physical activity per day on at least 7 days per week [19, 21, 23, 24, 26, 34]. Regarding sedentary behavior, studies analyzed this behavior as hours per day of television, computer/video game, and screen time use or sitting time; also, most studies defined excessive sedentary behavior as at least 3 h average per school day.

School bullying victimization was assessed using a questionnaire. While a limited number of the included studies used a validated bullying questionnaire [20, 22, 25, 29, 31, 32, 35], the majority of the studies used a single item to assess bullying (e.g., “During the past 12 months, have you ever been bullied on school property?”). Moreover, most studies focused on traditional bullying victimization (i.e. referring to physical abuse behaviors, verbal or weight-teasing, psychological abuse and social exclusion) and five in cyberbullying (i.e. texting, emails, social network sites) [22, 26, 30, 33, 34].

### Risk of bias within studies

All 18 studies met at least 6 criteria and were considered to have moderate methodological quality. The average score was 7.5/14.0 (Table 1 and Additional file 1).

### Meta-analysis of the association between physical activity and bullying victimization

**Fig. 2:**
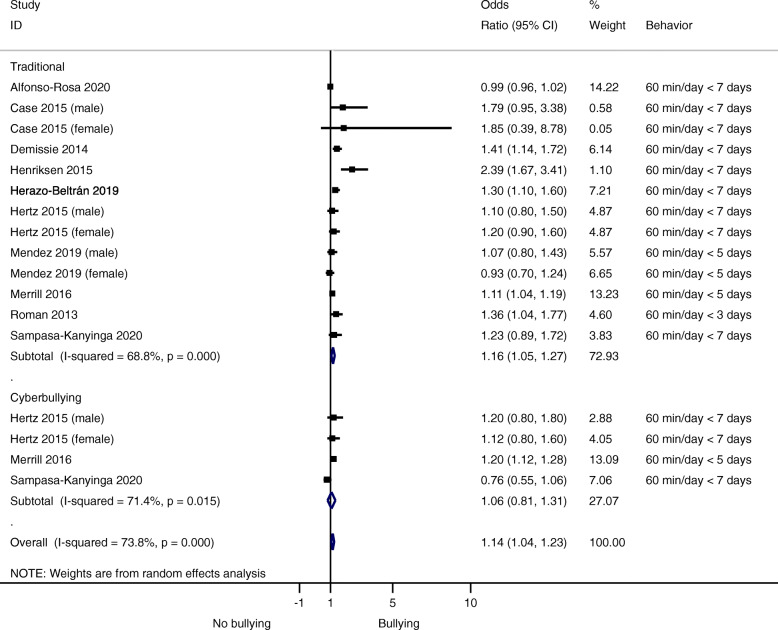
Forest plot of studies comparing bullying victimization between those who are inactive versus active peers. Traditional bullying: physical abuse behaviors, verbal, psychological abuse and social exclusion. Cyberbullying: texting, emails, and social network sites

Overall, there was evidence that not meeting the current physical activity guidelines was associated with higher bullying victimization (OR = 1.14, 95% CI, 1.04–1.23; *p* < 0.001; I^2^ = 73.8%) (Fig. 2).

According to the type of bullying, not meeting the physical activity guidelines was also associated with higher traditional bullying (OR = 1.16 95% CI, 1.05–1.27; *p* < 0.002; I^2^ = 68.8%) (Fig. 2). Finally, according to sex, not meeting the physical activity guidelines was not associated with bullying victimization in females (OR = 1.08, 95% CI, 0–91–1.28; *p* = 0.395; I^2^ = 0%) or males (OR = 1.15, 95% CI, 0.96–1.38; *p* = 0.126; I^2^ = 0%).

### Meta-analysis of the association between sedentary behavior and bullying victimization

**Fig. 3:**
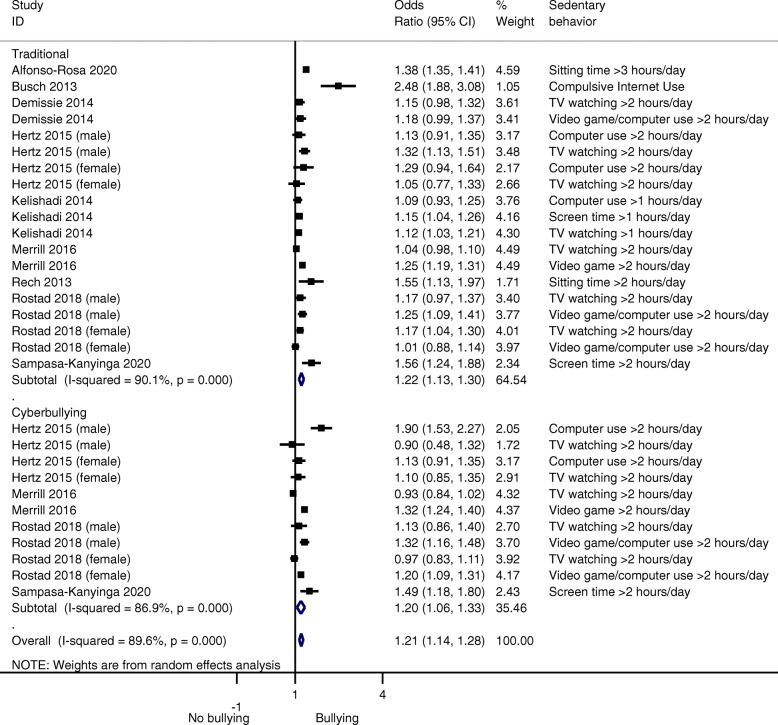
Forest plot of studies for bullying victimization between those who are excessive sedentary versus non-sedentary peers. Traditional bullying: physical abuse behaviors, verbal, psychological abuse and social exclusion. Cyberbullying: texting, emails, and social network sites

Excessive sedentary behavior was associated with higher bullying (OR = 1.21, 95% CI, 1.14–1.28; *p* < 0.001; I^2^ = 89.6%), cyberbullying (OR = 1.20, 95% CI, 1.06–1.33; *p* < 0.001; I^2^ = 86.9%), and traditional bullying (OR = 1.22, 95% CI, 1.13–1.30; *p* < 0.001; I^2^ = 90.1%) (Fig. 3). Results also showed associations in both sexes (males, OR = 1.25, 95% CI, 1.14–1.37; *p* < 0.001; I^2^ = 29.06%; females, OR = 1.10, 95% CI, 1.03–1.18; *p* = 0.006; I^2^ = 23.46%). According to type of sedentary behavior, watching TV (OR = 1.08, 95% CI, 1.01–1.14; *p* = 0.016; I^2^ = 51.16%), computer use (OR = 1.21, 95% CI, 1.04–1.42; *p* = 0.014; I^2^ = 50.32%), leisure time spent playing video games (OR = 1.27, 95% CI, 1.20–1.35; *p* < 0.001; I^2^ = 0%) or total sitting time (OR = 1.38, 95% CI, 1.34–1.42; *p* < 0.001; I^2^ = 0%) were associated with higher bullying victimization.

### Sensitivity analysis and publication bias

When the impact of individual studies was examined by removing studies from the analysis one at a time, we observed that the pooled results estimate remained consistent.

Both funnel plot asymmetry (Additional file 1) and Egger test showed no significant publication bias for physical activity (bias = 1.99; 95%CI, 0.38 to 3.60; *p* = 0.018) and sedentary behavior (bias = − 1.14; 95%CI, − 2.89 to 0.61; *p* = 0.194), indicating no evidence of publication bias.

## Discussion

To the best of our knowledge, the current review is the first to assess the associations of physical activity and sedentary behavior with bullying victimization among children and adolescents. Our study found that not meeting the physical activity guidelines and excessive sedentary behavior were associated with 14 and 21% higher bullying victimization, respectively. When looking at specific forms of bullying, we also found a consistent association between sedentary behavior with both traditional and cyberbullying.

### Physical activity and bullying victimization

Engaging in 1 h or more per day of moderate-to-vigorous physical activity is normally recommended for better health and quality of life among youths [7, 8]. The Physical Activity Guidelines for Americans recently suggested that meeting the physical activity recommendations is vital to the physical, psychological/social, and cognitive health of school-aged children and adolescents [7]. Our findings suggest that not meeting physical activity guidelines was associated with 14% higher bullying victimization among children and adolescents. This finding supports the work of other studies in this area [38, 39]. For example, a recent randomized controlled trial seems to corroborate the role of physical activity on bullying victimization among Chilean children [39].

Several factors may underpin the relationship between physical inactivity and bullying victimization: (a) bullying victimization occurs frequently in activities not closely supervised and therefore youth tend to avoid these activities [40, 41]; and (b) physically inactive youth may be at an increased risk of being bullied due to factors such as poor motor skills [42], physical fitness [43], and low self-confidence to engage in physical activities.

### Sedentary behavior and bullying victimization

Among children and adolescents, television viewing and other screen-based forms of entertainment are the most prevalent leisure-time sedentary behaviors [44]. Recent studies of electronic media use and TV viewing have concluded that this type of sedentary behavior (i.e. screen-based) is associated with lower pro-social behavior, self-esteem [45] and subjective well-being [13] in children and adolescents. In this regard, our meta-analysis reveals that excessive sedentary behavior was related with 21% higher bullying victimization, and found that this relationship is consistent across both, traditional (22%) and cyberbullying (20%). Increased screen-based activities use may diminish experiences of personal interaction that helps youth develop interpersonal relationships and academic skills, pro-social behavior and conflict resolution [46], which could place youths at risk of multiple forms of victimization in relationships with dating partners, friends, and peers [47]. Furthermore, studies suggest that friendships can function as a protective factor against the negative adjustment often experienced by bullied youth [47]. In contrast, screen-based activities favor loneliness [12]. For example, more screen time such as play video game seems to compromise youth development of interpersonal skills hence making them vulnerable to all forms of bullying [33]. Regarding electronic bullying victimization, both male and female youths could be exposed to cyberbullying through social media consuming during computer use [33].

Socio-economic factors seem to be related with bullying prevalence. For example, it has been suggested that adolescents from lower social classes are more exposed to victimization compared to adolescents from high socio-economic status [24]. In this regard, it is interesting to highlight findings from Alfonso-Rosa et al. [19] who indicated that the odds of being bullied were lower for adolescents who exceeded sitting guidelines in high Human Development Index countries compared to those in low Human Development Index countries.

### Limitations

There are several limitations to this meta-analysis that should be acknowledged. First, our results are based on cross-sectional studies and so causality cannot be inferred. Reverse causality could also be true, i.e., youths bullied could be less prone to follow healthy lifestyle habits [48, 49]. Second, all of the studies except two [22, 36] did not include a rigorous physical activity and sedentary behavior assessment. Therefore, self-reported questionnaires could potentially be subject to socially desirable reporting bias. Third, only five studies used validated bullying questionnaires [20, 22, 29, 31, 32, 35]. In this sense, outcome measures for bullying are not standardized across the studies performed in different countries with likely variable cultural norms. Also, studies used different time frames to determine bullying (i.e. past month, past couple of months, current or past school year). Thus, the discrepancy in definitions might explain the heterogeneity of our results. Finally, physical activity and sedentary behavior were dichotomized, therefore, the current study cannot determine if the association varies across different levels of physical activity or sedentary intensity and participation.

## Conclusions

This meta-analysis establishes the first quantitative framework for understanding the influence of physical activity on bullying victimization, showing that not meeting the physical activity guidelines and excessive sedentary behavior are associated with higher bullying victimization. Furthermore, it lays the groundwork for future studies and interventions aimed to bullying victimization prevention through promoting physical activity and reducing sedentary behaviors.

## Supplementary information

**Additional file 1.** Table. Items of Quality Assessment Tool for Observational Cohort and Cross-sectional studies. Figure. Funnel plot for physical activity on bullying victimization, with 95% confidence limits. Figure. Funnel plot for sedentary behavior on bullying victimization, with 95% confidence limits.

## Data Availability

The datasets used and/or analyzed during the current study are available from the corresponding author on reasonable request.

## Abbreviations

CDC: Centers for disease control and prevention
CI: Confidence interval
TV: Television
OR: Odds ratio
