# Supplementary material for: Bullying victimization, physical inactivity and sedentary behavior among children and adolescents: a meta-analysis
Source: Int J Behav Nutr Phys Act. 2020 Sep 11;17:114. doi: 10.1186/s12966-020-01016-4 (PMC7488515; doi:10.1186/s12966-020-01016-4)
Supplement: Supplementary file 1 — Additional file 1. Table. Items of Quality Assessment Tool for Observational Cohort and Cross-sectional studies. Figure. Funnel plot for physical activity on bullying victimization, with 95% confidence limits. Figure. Funnel plot for sedentary behavior on bullying victimization, with 95% confidence limits. [file 12966_2020_1016_MOESM1_ESM.docx]

**Additional file**

Search strategy

Physical activity: (((((((((((((((((((((((("light intensity physical activity"[Title/Abstract]) OR "moderate intensity physical activity"[Title/Abstract]) OR "vigorous intensity physical activity"[Title/Abstract]) OR "counts per minute"[Title/Abstract]) OR "cpm"[Title/Abstract]) OR "energy expenditure"[Title/Abstract]) OR "locomotor activity"[Title/Abstract]) OR "physical activity"[Title/Abstract]) OR "physical activity intensity"[Title/Abstract]) OR "physical activity questionnaire"[Title/Abstract]) OR "physical load"[Title/Abstract]) OR "physical movement"[Title/Abstract]) OR "physical activity guidelines"[Title/Abstract]) OR "physical activity recommendations"[Title/Abstract]) OR "regular physical activity"[Title/Abstract]) AND "bullying"[Title/Abstract]) OR "victimization"[Title/Abstract]) OR "peer relation"[Title/Abstract])

Sedentary behaviour: ((((((((((((((((((("physical inactivity"[Title/Abstract]) OR "low energy expenditure"[Title/Abstract]) OR "physically inactive"[Title/Abstract]) OR "computer"[Title/Abstract]) OR "video game"[Title/Abstract]) OR "smartphone"[Title/Abstract]) OR "tablet"[Title/Abstract]) OR "internet"[Title/Abstract]) OR "screen"[Title/Abstract]) OR "screen based"[Title/Abstract]) OR "television"[Title/Abstract]) OR "tv"[Title/Abstract]) OR "sitting"[Title/Abstract]) OR "sedentary"[Title/Abstract]) OR "lying"[Title/Abstract]) OR "sedentary behavior"[Title/Abstract]) OR "sedentary behaviour"[Title/Abstract])) AND (((chile[Title/Abstract]) AND ((((((((("child"[Title/Abstract]) OR "children"[Title/Abstract]) OR "school-aged"[Title/Abstract]) OR "teen"[Title/Abstract]) OR "adolescent"[Title/Abstract]) OR "young person"[Title/Abstract]) OR "youth"[Title/Abstract]) OR "kid"[Title/Abstract]) OR "juvenile"[Title/Abstract]) AND "bullying"[Title/Abstract]) OR "victimization"[Title/Abstract]) OR "peer relation"[Title/Abstract])

**Table**

| **Items of Quality Assessment Tool for Observational Cohort and Cross-sectional studies** | | | | | | | | | | | | | | | |
| --- | --- | --- | --- | --- | --- | --- | --- | --- | --- | --- | --- | --- | --- | --- | --- |
| Author | 1 | 2 | 3 | 4 | 5 | 6 | 7 | 8 | 9 | 10 | 11 | 12 | 13 | 14 | Total score |
| Alfonso-Rosa 2020 | 🗸 | 🗸 | 🗸 | 🗸 | 🗴 | NA | NA | 🗸 | 🗸 | NA | 🗸 | NA | NA | 🗸 | 8 |
| Busch 2013 | 🗸 | 🗸 | NR | 🗸 | 🗴 | NA | NA | 🗸 | 🗸 | NA | 🗸 | NA | NA | 🗸 | 7 |
| Case 2015 | 🗸 | 🗸 | 🗴 | 🗸 | 🗴 | NA | NA | 🗸 | 🗸 | NA | 🗸 | NA | NA | 🗸 | 7 |
| Corral-Pernía 2017 | 🗸 | 🗸 | NR | 🗸 | 🗴 | NA | NA | 🗸 | 🗸 | NA | 🗸 | NA | NA | 🗴 | 6 |
| Demissie 2014 | 🗸 | 🗸 | 🗸 | 🗸 | 🗴 | NA | NA | 🗸 | 🗸 | NA | 🗸 | NA | NA | 🗸 | 8 |
| Henriksen 2015 | 🗸 | 🗸 | 🗸 | 🗸 | 🗴 | NA | NA | 🗸 | 🗸 | NA | 🗸 | NA | NA | 🗸 | 8 |
| Herazo-Berltrán 2019 | 🗸 | 🗸 | NR | 🗸 | 🗴 | NA | NA | 🗸 | 🗸 | NA | 🗸 | NA | NA | 🗸 | 7 |
| Hertz 2015 | 🗸 | 🗸 | 🗸 | 🗸 | 🗴 | NA | NA | 🗸 | 🗸 | NA | 🗸 | NA | NA | 🗸 | 8 |
| Katapally 2018 | 🗸 | 🗸 | 🗸 | 🗸 | 🗴 | NA | NA | 🗸 | 🗸 | NA | 🗸 | NA | NA | 🗸 | 8 |
| Kelishadi 2014 | 🗸 | 🗸 | 🗸 | 🗸 | 🗴 | NA | NA | 🗸 | 🗸 | NA | 🗸 | NA | NA | 🗸 | 8 |
| Mendez 2019 | 🗸 | 🗸 | NR | 🗸 | 🗸 | NA | NA | 🗸 | 🗸 | NA | 🗸 | NA | NA | 🗴 | 7 |
| Merrill and Hanson 2016 | 🗸 | 🗸 | 🗸 | 🗸 | 🗴 | NA | NA | 🗸 | 🗸 | NA | 🗸 | NA | NA | 🗸 | 8 |
| Rech 2013 | 🗸 | 🗸 | 🗸 | 🗸 | 🗸 | NA | NA | 🗸 | 🗸 | NA | 🗸 | NA | NA |  | 8 |
| Roman 2013 | 🗸 | 🗸 | 🗸 | 🗸 | 🗴 | NA | NA | 🗸 | 🗸 | NA | 🗸 | NA | NA | 🗸 | 8 |
| Rostad 2018 | 🗸 | 🗸 | 🗸 | 🗸 | 🗴 | NA | NA | 🗸 | 🗸 | NA | 🗸 | NA | NA | 🗸 | 8 |
| Sampasa-Kanyinga 2020 | 🗸 | 🗸 | 🗸 | 🗸 | 🗴 | NA | NA | 🗸 | 🗸 | NA | 🗸 | NA | NA | 🗸 | 8 |
| Storch 2007 | 🗸 | 🗸 | 🗸 | 🗸 | 🗴 | NA | NA | 🗸 | 🗸 | NA | 🗸 | NA | NA | 🗴 | 7 |
| Watanabe 2017 | 🗸 | 🗸 | 🗸 | 🗸 | 🗴 | NA | NA | 🗸 | 🗸 | NA | 🗸 | NA | NA | 🗴 | 7 |
| 🗸 indicates “yes”, 🗴 indicates “no”, and NR indicates “not reported”.  NA: not applicable due to the cross-sectional design of the studies. | | | | | | | | | | | | | | | |

Figure. Funnel plot for physical activity on bullying victimization, with 95% confidence limits

Figure. Funnel plot for sedentary behavior on bullying victimization, with 95% confidence limits
